# Inhibition of transforming growth factor β-activated kinase 1 prevents inflammation-related cartilage degradation in osteoarthritis

**DOI:** 10.1038/srep34497

**Published:** 2016-09-29

**Authors:** Jin Cheng, Xiaoqing Hu, Linghui Dai, Xin Zhang, Bo Ren, Weili Shi, Zhenlong Liu, Xiaoning Duan, Jiying Zhang, Xin Fu, Wenqing Chen, Yingfang Ao

**Affiliations:** 1 Institute of Sports Medicine, Beijing Key Laboratory of Sports Injuries, Peking University Third Hospital, Beijing 100191, China

## Abstract

Osteoarthritis (OA) is a common debilitating joint disorder, there’s still no available disease-modifying drug for OA currently. This study aims to explore the role of TAK1 in OA pathogenesis and therapeutic efficiency of TAK1 inhibition for OA. The contribution of TAK1 to OA pathogenesis was investigated by intra-articular injection of TAK1-encoding adenovirus in rats. TAK1 inhibitor 5Z-7-induced expression changes of extracellular matrix (ECM)-related genes were detected by real-time PCR. The protective effect of 5Z-7 against OA progression was evaluated in a post-traumatic OA rat model. Our results showed that intra-articular injection of Ad-*Tak1* induced cartilage destruction and OA-related cytokine secretion in rat joints. TAK1 inhibition by 5Z-7 efficiently blocked NF-κB, JNK and p38 pathways activation in OA chondrocytes and synoviocytes, Meanwhile, 5Z-7 significantly decreased the expression of matrix-degrading enzymes and pro-inflammatory cytokine, while increased ECM protein expression, which are all crucial components in OA. 5Z-7 also ameliorated ECM loss in OA cartilage explants. More importantly, 5Z-7 significantly protected against cartilage destruction in a rat model of OA. In conclusion, our findings provide the first *in vivo* evidence that TAK1 contributes to OA by disrupting cartilage homeostasis, thus represents an ideal target for OA treatment, with 5Z-7 as a candidate therapeutic.

Osteoarthritis (OA) is a common degenerative joint disorder, in which a variety of aetiological risk factors and pathophysiological processes contribute to its progressive nature. OA is primarily characterized by cartilage destruction, which are mainly contributed by two pathways: upregulation of matrix degradation enzymes and downregulation of cartilage-specific extracellular matrix (ECM) proteins^1^. Ever since Pelletier *et al*. reconceptualized OA as an inflammatory disease of diarthrodial synovial joints, emerging evidences have shown that immune-related inflammation response is also a key contributor to OA joint pathology^2^. Nevertheless, despite the fact that elevated levels of pro-inflammatory cytokines such as IL-1, TNF-α and IL-6 promote articular cartilage ECM protein degradation or synergize with other cytokines to amplify and accelerate cartilage destruction, clinical trials employing several anti-IL-1 strategies have generally been unsuccessful due to lack of significant clinical efficacy^3^,^4^. Therefore, novel approaches may need to take advantage of signaling pathways that are likely to govern these cytokines expression in OA joint.

The production of pro-inflammatory cytokines is mainly mediated by the nuclear factor-kappaB (NF-κB) pathway and mitogen-activated protein kinases (MAPKs) pathway, which are often aberrantly activated during OA^5^,^6^,^7^. Transforming growth factor β-activated kinase 1 (TAK1) is a member of the MAPK kinase kinase (MAP3K) family, it is known to control diverse functions ranging from innate and adaptive immune system activation to vascular development and apoptosis through activating downstream effectors including NF-κB and MAPKs^8^,^9^,^10^. Studies using a number of genetically engineered mice have uncovered an indispensable role of TAK1 for maintenance of tissue homeostasis in a variety of organs^11^,^12^,^13^,^14^. Numerous previous reports have also indicated that TAK1 is tightly related to cartilage homeostasis: mice with conditional deletion of *Tak1* in cartilage displayed severe chondrodysplasia and joint abnormalities including elbow dislocation and tarsal fusion, indicating an essential role of TAK1 in the morphogenesis and maintenance of cartilage^15^. On the other hand, studies have also shown that silencing TAK1 using specific siRNA in a human chondrosarcoma cell line reduces IL-1β-induced upregulation of MMP1, MMP13 and TNF-α^16^, and TAK1 mediates COX2 expression and PGE2 release in synoviocytes and chondrocytes^17^,^18^, suggesting that TAK1 might be involved in OA-related pain. The above findings indicated that deregulation of TAK1 might disrupt cartilage homeostasis and therefore provoke tissues damage, however, the specific role TAK1 plays in OA pathogenesis still remains to be clarified, and systematic biological effects of TAK1 inhibition in OA as well as its potential therapeutic benefits are still poorly understood.

In the present study, we report that intra-articular overexpression of TAK1 leads to typical OA pathological changes in rat knees, and TAK1 inhibition by a small molecular inhibitor 5Z-7 protects against osteoarthritic cartilage degradation and synovial inflammation via regulating a series of ECM-related genes. These results suggest that TAK1 is an attractive therapeutic target in OA and its inhibitor 5Z-7 presents a promising candidate for OA drug development.

## Results

### Intra-articular TAK1 overexpression causes experimental OA cartilage destruction

**Figure 1:**
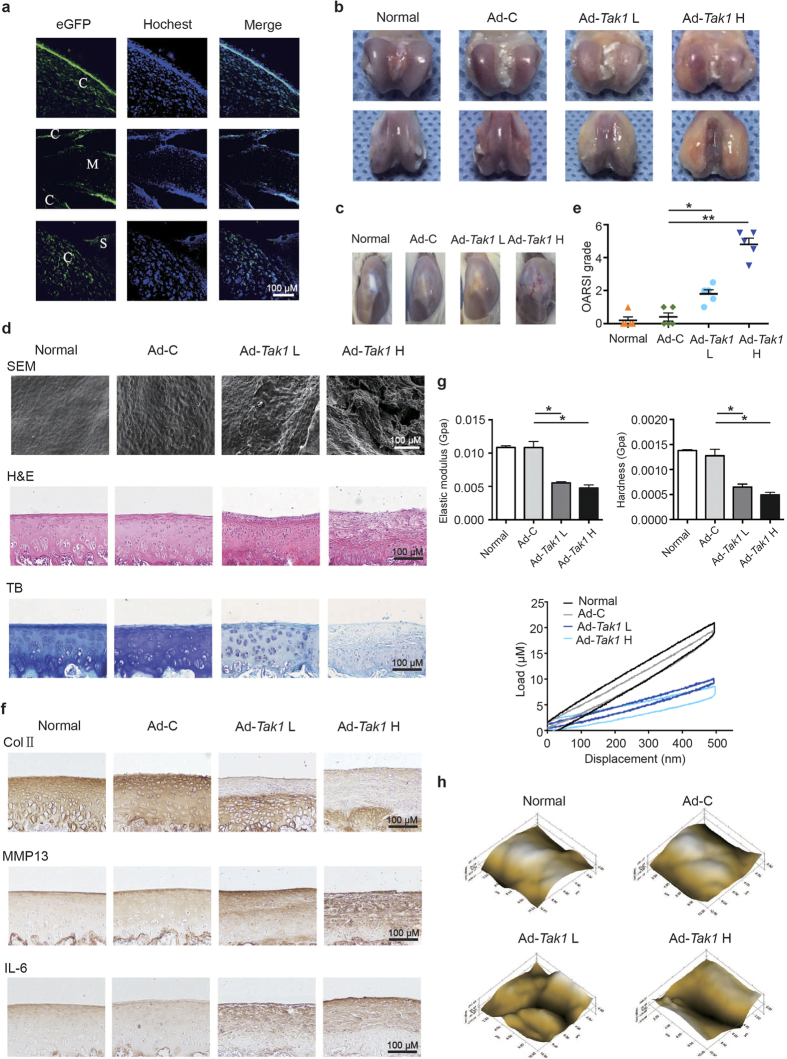
Intra-articular overexpression of TAK1 causes cartilage destruction in rats. (**a**) Representative immunofluorescence image of cartilage sections from rats intra-articularly injected with Ad-*Tak1*. Green: GFP, blue: DAPI. C: cartilage, M: meniscus, S: synovium. Scale bar, 100 μm. (**b**) Representative images of rat articular cartilage from normal, Ad-C, Ad-*Tak1* L and Ad-*Tak1* H groups. (**c**) Representative gross appearance of rat joints from each group. (**d**) SEM, H&E and toluidine blue staining of articular cartilage from each group. Scale bar, 100 μm. (**e**) OARSI scores of articular cartilage from each group. *P < 0.05, **P < 0.01. (**f**) IHC staining of type II collagen, MMP13 and IL-6 of articular cartilage from each group. Scale bar, 100 μm. (**g**) The biomechanical properties including elastic modulus, hardness and load-displacement curves of cartilage surface from each group. *P < 0.05. (**h**) Microscopic geomorphology of cartilage surface from each group.

The contribution of TAK1 to OA pathogenesis was examined by intra-articular overexpression of TAK1 in rats, since delivery of recombinant protein is often therapeutically limited by their short half-life, we chose to use viral gene transfer approach. To evaluate sub-tissue distribution of the adenovirus, frozen sections of rat knee joints were examined by confocal microscopy 24 hours after injection of green fluorescent protein (GFP)-tagged Ad-*Tak1*, as shown in Fig. 1a, GFP fluorescence was observed in cartilage, synovium, as well as meniscus, indicating that Ad-*Tak1* was able to penetrate and efficiently transduce cells in the above three tissue types.

Next, we sought to determine whether TAK1 overexpression in rat joints was sufficient enough to induce OA. Macroscopically, significant cartilage hypertrophy, synovium hyperplasia, as well as angiogenesis were observed in Ad-*Tak1* injection groups in a dose dependent manner one month after injection (Fig. 1b,c). Microscopically, cartilage surfaces in Ad-*Tak1* injection groups were much rougher than healthy group and Ad-control injection group as shown by the scanning electron micrograph (SEM) (Fig. 1d). Cartilage destruction condition was examined using hematoxylin-eosin (H&E) and toluidine blue staining, and then scored using the Osteoarthritis Research Society International (OARSI) grading system. The results revealed that TAK1 overexpression caused significant degeneration of articular cartilage relative to control groups (Fig. 1d,e). IHC analysis showed that TAK1 overexpression caused downregulation of type II collagen, with significantly elevated level of matrix degradation enzyme MMP13 and pro-inflammatory cytokine IL-6 in rat cartilage (Fig. 1f). The expression efficiency of TAK1 in cartilage after virus injection was also confirmed by IHC (Supplementary Figure S1).

Then we performed nanoindentation assay to detect biomechanical properties of the cartilage surfaces in each group. Compared to control groups, two Ad-*Tak1* injection groups both exhibited significant lower elastic modulus and hardness, and the load-displacement curves further revealed the impaired biomechanical strength of cartilage caused by TAK1 overexpression (Fig. 1g). Moreover, the microscopic geomorphology of the indentation zones in Ad-*Tak1* injection groups appeared to be much scraggier and lumpier than control groups (Fig. 1h). Taken together, the above data suggested that TAK1 overexpression promotes OA development by inducing cartilage degradation.

### TAK1 induces secretion of OA-related cytokines in synovial fluid

**Figure 2:**
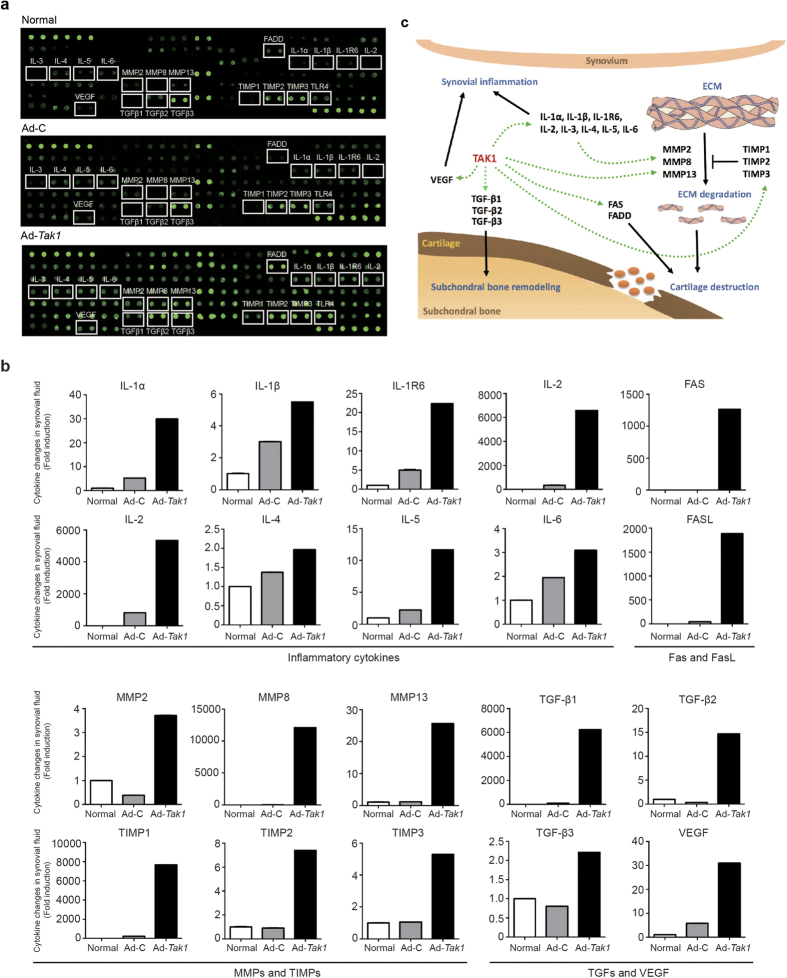
Intra-articular TAK1 overexpression upregulated the expression of OA-related cytokines in rats’ synovial fluid. Cytokine expression in the synovial fluid from normal, Ad-C and Ad-*Tak1* groups was analyzed using biotin label-based rat antibody array (RayBio, AAR-BLG-1). (**a**) Representative microarray images of each group. (**b**) The normalized expression changes of OA-related cytokines in synovial fluid of each group based on the microarray result. (**c**) The network regulation of the aberrantly upregulated cytokines and their contribution to OA pathogenesis.

To further investigate the pathological changes TAK1 caused in the knee joint, the expression levels of cytokines in synovial fluid from healthy group, Ad-control group and Ad-*Tak1* (high dose) group were evaluated using a rat antibody array consisted of 90 rat proteins including cytokines, chemokines, growth factors, angiogenic factors, etc. The array results showed that the secretion of numerous classic OA-related genes was induced by TAK1, including interleukins, MMPs, TGF-βs, vascular endothelial growth factor (VEGF), death domain complex (FAS and FADD) (Fig. 2a,b). Figure 2c illustrated how the network activity initiated by TAK1 contributes to OA pathogenesis inside the articular cavity. Briefly, the upregulation of VEGF promotes synovial angiogenesis and subsequent immune-related inflammation response together with interleukins. On the other hand, ECM degradation caused by elevated level of MMPs, subchondral bone remodeling mediated by TGF-β, as well as chondrocytes apoptosis induced by FAS and FADD synergistically lead to cartilage destruction (Fig. 2c). Collectively, these data revealed that TAK1 activation increased OA-related cytokine secretion in synovial fluid inside the joint.

### TAK1 inhibition by 5Z-7 suppressed NF-κB, JNK and p38 pathways activation in OA chondrocytes and synoviocytes

**Figure 3:**
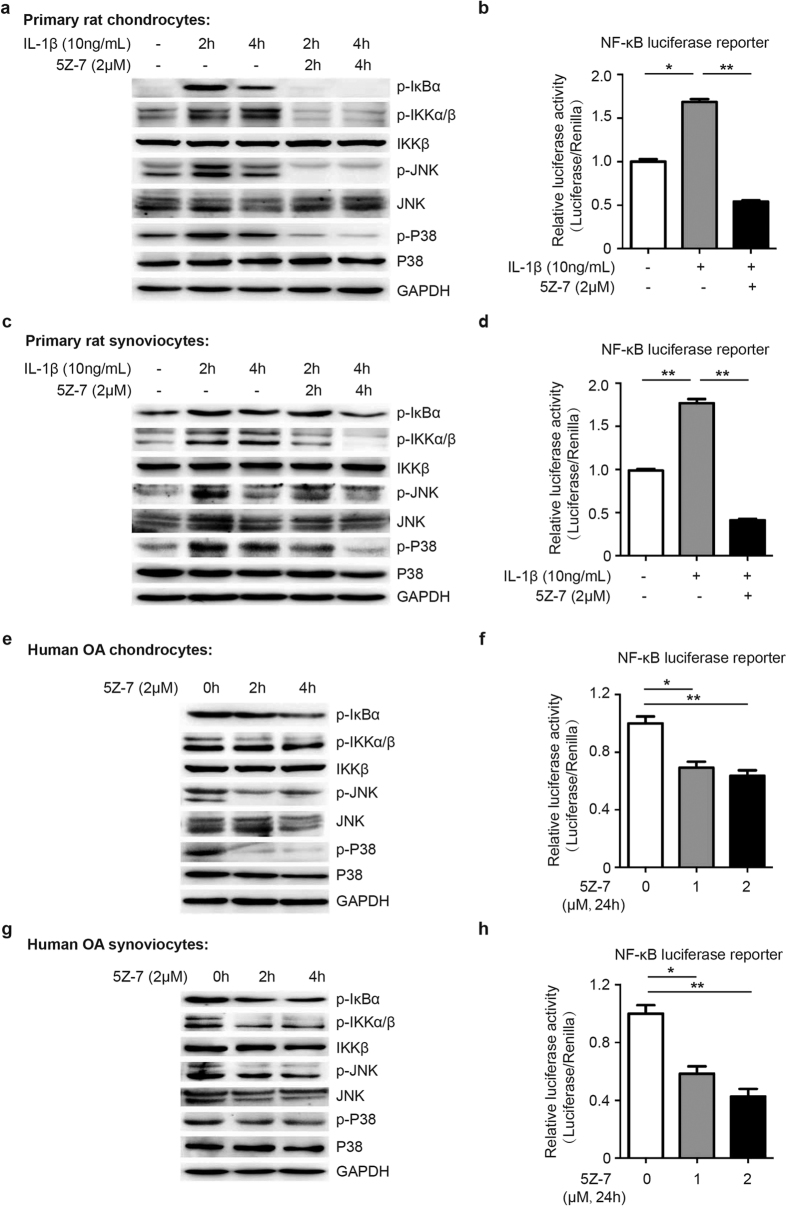
TAK1 inhibitor 5Z-7 suppresses NF-κB, JNK and p38 activation in OA chondrocytes and synoviocytes. (**a,b**) Detection of the activation level of NF-κB, JNK and p38 pathways by (**a**) western blotting and (**b**) luciferase reporter assay in primary rat chondrocytes treated with IL-1β with or without 5Z-7 at indicated time points. (**c,d**) Detection of the activation level of NF-κB, JNK and p38 pathways by (**c**) western blotting and (**d**) luciferase reporter assay in primary rat synoviocytes treated with IL-1β with or without 5Z-7 at indicated time points. (**e,f**) Detection of the activation level of NF-κB, JNK and p38 pathways by (**e**) western blotting and (**f**) luciferase reporter assay in human OA chondrocytes treated with 5Z-7. (**g**,**h**) Detection of the activation level of NF-κB, JNK and p38 pathways by (**g**) western blotting and (**h**) luciferase reporter assay in human OA synoviocytes treated with 5Z-7. *P < 0.05, **P < 0.01.

Since TAK1 is known as a pivotal intermediate for the activation of NF-κB, JNK and p38 MAPK pathways in various cell types^19^,^20^, we examined that if pharmacological inhibition of TAK1 activity using 5Z-7, a small molecular inhibitor of TAK1, could block the activation of the above pathways in chondrocytes and synoviocytes, two major cell types in articular cavity. We first conducted IL-1β treatment to establish an OA-like cell model for *in vitro* study^21^. Western blot results showed that 5Z-7 completely blocked IL-1β-induced IKK and IκBα phosphorylation, as well as JNK and p38 phosphorylation in primary rat chondrocytes (Fig. 3a). Moreover, luciferase reporter assay showed that 5Z-7 also inhibited IL-1β-induced transcription activity of NF-κB responsive element in primary rat chondrocytes (Fig. 3b). On the other hand, the same inhibitory effect of 5Z-7 was also seen in primary rat synoviocytes (Fig. 3c,d).

There are growing evidences suggesting that NF-κB and MAPK pathways tend to be over-activated during OA progression^6^,^7^. Therefore, we investigated whether TAK1 inhibition by 5Z-7 could suppress the basal activation level of these two pathways in chondrocytes harvested from OA patients. As expected, western blot results suggested that basal phosphorylation level of IKK, IκBα, JNK and p38 were higher in OA chondrocytes than normal chondrocytes, which was consistent with previous reports, and 5Z-7 significantly reduced their phosphorylation level (Fig. 3e). Furthermore, 5Z-7 also inhibited the basal transcription activity of NF-κB responsive element in OA chondrocytes and synoviocytes (Fig. 3f). On the other hand, 5Z-7 exhibited the same suppressive function in OA synoviocytes (Fig. 3g,h). Additionally, CCK-8 results revealed that 5Z-7 had no significant cytotoxity on chondrocytes and synoviocytes (Supplementary Figure S2). The above results suggested that pharmacological inhibition of TAK1 by 5Z-7 suppressed not only IL-1β-induced activation of NF-κB, JNK and p38 MAPK pathways in normal chondrocytes and synoviocytes, but also basal activation of these pathways in those cells under OA condition.

### TAK1 inhibition regulated ECM-related genes expression in OA chondrocytes and synoviocytes

We next explored the impact of TAK1 inhibition on the expression pattern of a series of ECM-related genes in primary rat chondrocytes. The results showed that IL-1β treatment significantly upregulated the expression of MMP1, MMP13, ADAMTS5 and pro-inflammatory cytokine IL-6, while downregulated Aggrecan and COL2A1, as well as chondrogenesis specific marker SOX9, without significant impact on ADAMTS1 expression, while 5Z-7 greatly suppressed, and in some cases reversed the above effect (Supplementary Figure S3a).

During OA development, inflammatory synovium is the main producer of matrix-degrading enzymes and pro-inflammatory cytokines, so we also detected the impact of 5Z-7 on the expression of the above genes in rat synoviocytes. As shown in Supplementary Figure S3b, IL-1β treatment induced the expression of MMP1, MMP13, ADAMTS1, ADAMTS5, and IL-6 in synoviocytes to a greater extent compared to that in chondrocytes, once again emphasize the importance of synovium in OA development, and 5Z-7 suppressed the effect of IL-1β, which was also more significant than that in chondrocytes.

**Figure 4:**
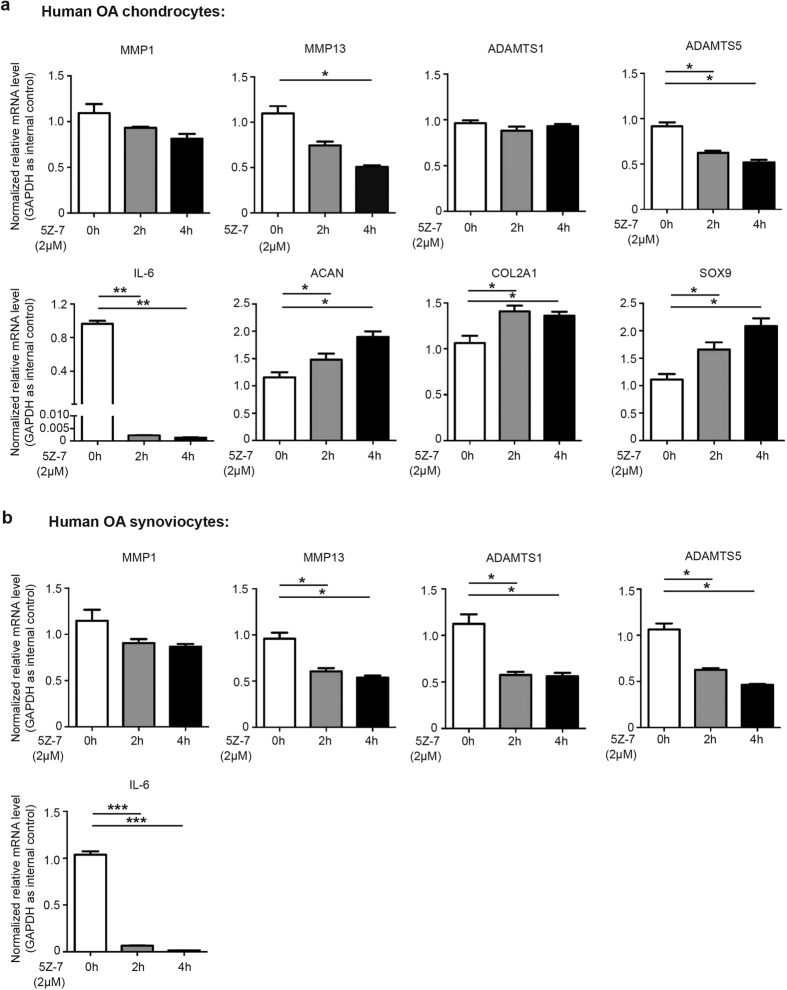
TAK1 inhibitor 5Z-7 regulated the expression of ECM-related genes in OA chondrocytes and synoviocytes. (**a,b**) The mRNA expression level of several classic ECM-related genes in (**a**) human OA chondrocytes and (**b**) human OA synoviocytes treated with 5Z-7 by real-time PCR. *P < 0.05, **P < 0.01, ***P < 0.001.

Since IL-1β treatment could not completely reflect the real case of OA, we proceeded to investigate whether 5Z-7 could modulate the expression of ECM-related genes in chondrocytes derived from OA patients. As expected, 5Z-7 downregulated the expression of MMP13, ADAMTS5 and IL-6, and upregulated the expression of Aggrecan, COL2A1 and SOX9 in OA chondrocytes (Fig. 4a). As in OA synoviocytes, 5Z-7 significantly downregulated MMP13, ADAMTS1, ADAMTS5 and IL-6 expression levels (Fig. 4b). The above data indicated that pharmaceutical inhibition of TAK1 by 5Z-7 converted OA chondrocytes and synoviocytes into a healthier status by modulating a series of ECM-related genes.

### TAK1 inhibition ameliorated microenvironment of the co-culture system

**Figure 5:**
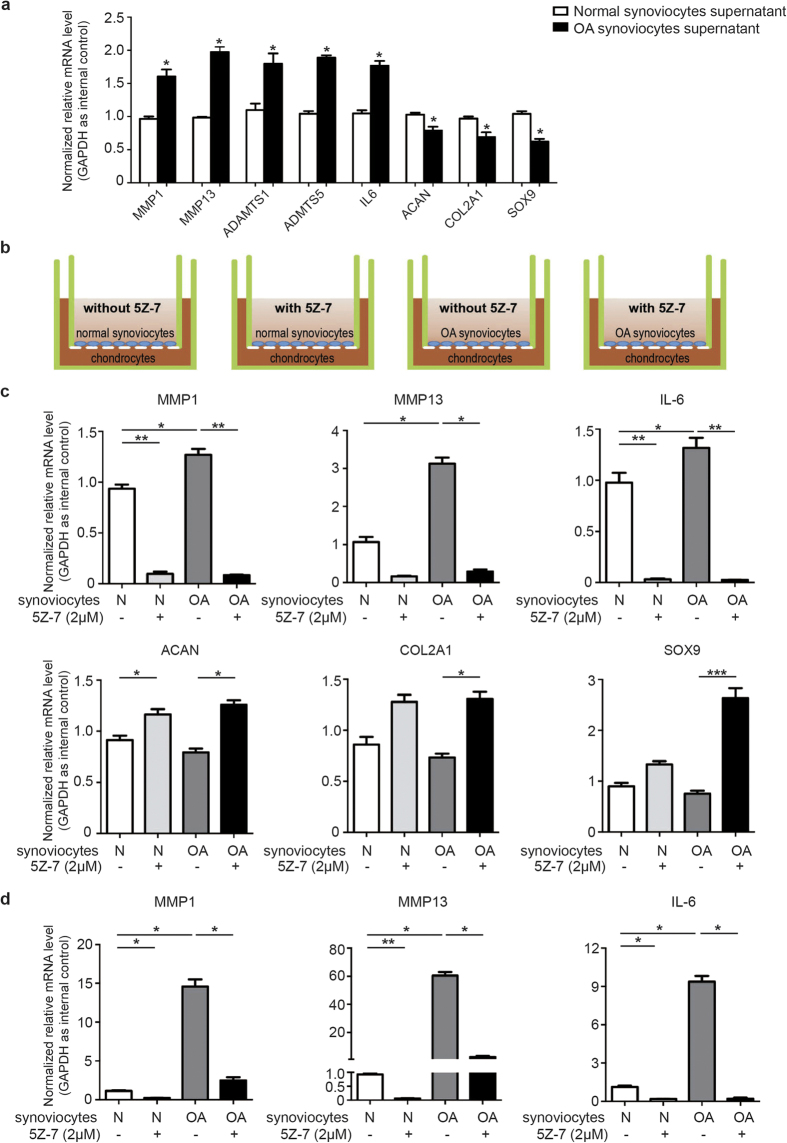
Inhibition of TAK1 modulated microenvironment homeostasis of the co-culture system. (**a**) The mRNA expression level of classic ECM-related genes in human chondrocytes treated with the supernatant from cultured normal or OA synoviocytes. (**b**) The schematic diagram of the co-culture system consisted of chondrocytes and synoviocytes with or without 5Z-7. (**c,d**) The mRNA expression level of classic ECM-related genes in (**c**) chondrocytes and (**d**) synoviocytes within the co-culture system after 24 hrs. *P < 0.05, **P < 0.01, ***P < 0.001.

OA is a systematic disease that involves pathological changes of the whole joint, during which the microenvironment is often disturbed and inside tissues including cartilage and synovium interact with each other to accelerate disease progression via autocrine and paracrine signaling^22^. In accordance with this, our preliminary results have also shown that OA synoviocytes culture supernatant significantly altered ECM-related genes expression pattern in normal chondrocytes (Fig. 5a), suggesting the cytokines OA synoviocytes secreted can convert chondrocytes into an OA-like pathological status. Next, we used a co-culture system consisted of human chondrocytes and synoviocytes to better imitate *in vivo* microenvironment of the articular cavity. We co-cultured normal chondrocytes with normal or OA synoviocytes with or without 5Z-7 treatment as indicated in Fig. 5b. After 24 hours of co-culture, we noticed that compared to normal synoviocytes, co-culture with OA synoviocytes significantly enhanced the expression of MMP1, MMP13 and IL-6 in chondrocytes, which was in turn suppressed by 5Z-7, and that 5Z-7 had a greater potency in chondrocytes co-cultured with OA synoviocytes (Fig. 5c). Furthermore, 5Z-7 increased the expression level of Aggrecan, COL2A1 and SOX9 in chondrocytes, also to a greater extent in those co-cultured with OA synoviocytes (Fig. 5c). As for synoviocytes in the co-culture system, 5Z-7 exhibited its inhibitory effect on MMP1, MMP13 and IL-6 expression both in normal groups and OA groups (Fig. 5d). The result of co-culturing for 48 hours has the same tendency as 24 hours (Supplementary Figure S4a,b). These results suggested that TAK1 inhibition by 5Z-7 ameliorated microenvironment homeostasis in the co-culture system consisted of chondrocytes and synoviocytes by regulating ECM-related genes expression.

### Application of TAK1 inhibitor prevents development of post-traumatic OA

**Figure 6:**
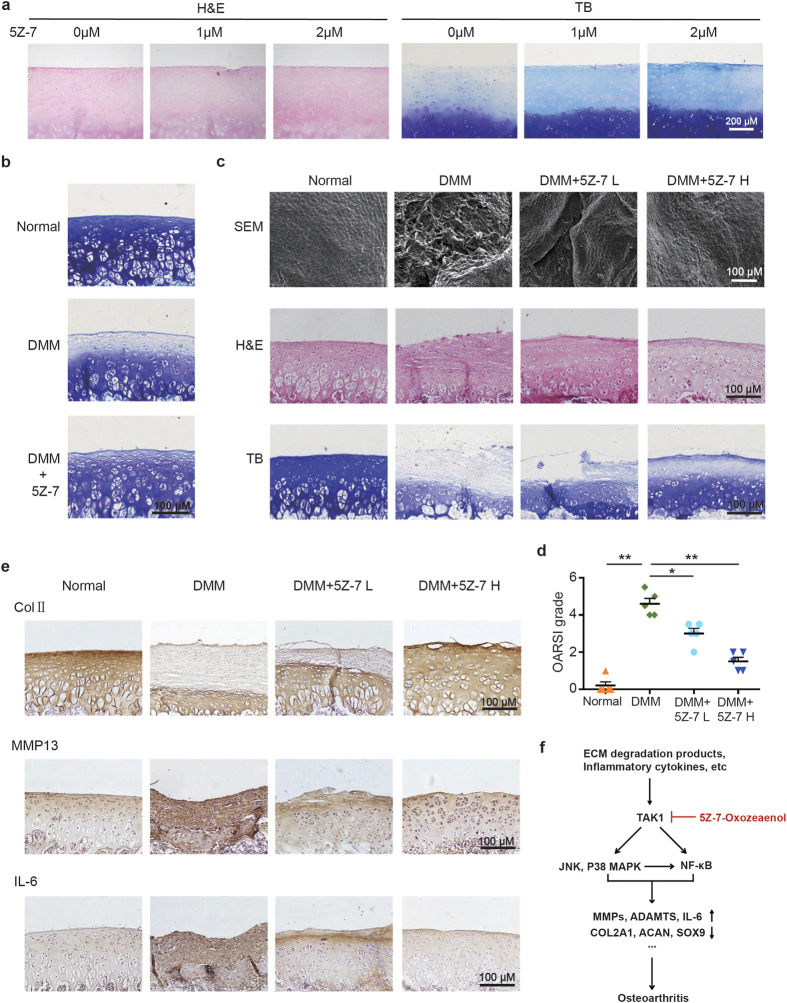
Application of 5Z-7 protects against OA development in a rat model induced by DMM. (**a**) H&E and toluidine blue staining of human OA cartilage explant treated with 5Z-7. Scale bar, 200 μm. (**b**) Toluidine blue staining of the articular cartilage from normal, DMM and DMM + 5Z-7 group (10 days after DMM surgery). Scale bar, 100 μm. (**c**) The SEM, H&E and toluidine blue staining of the articular cartilage from normal, DMM, DMM + 5Z-7 L and DMM + 5Z-7 H group (one month after DMM surgery). Scale bar, 100 μm. (**d**) OARSI scores of the articular cartilage from each group. *P < 0.05, **P < 0.01. (**e**) IHC staining of type II collagen, MMP13 and IL-6 of the cartilage from each group. Scale bar, 100 μm. (**f**) The working model of how pharmacological TAK1 inhibition protects against OA development.

Next, we proceeded to investigate the effect of TAK1 inhibition on human cartilage catabolism. Human OA cartilage explants were cultured in the presence or absence of 5Z-7 for a total of 10 days. The toluidine blue staining results showed that ECM loss in OA cartilage explants was gradually rescued by increasing doses of 5Z-7 (Fig. 6a), which further confirmed that TAK1 is involved in cartilage degradation during OA.

Finally, we applied destabilization of the medial meniscus (DMM) surgery to rats to ascertain whether TAK1 inhibition protects against post-traumatic OA development^23^,^24^. Our preliminary results showed that rats exhibited moderate loss of ECM in cartilage 10 days after DMM surgery as indicated by toluidine blue staining, while intra-articular injection of 5Z-7 significantly reversed that loss (Fig. 6b). As for one month after surgery, SEM of cartilage surfaces revealed that the joints of DMM rats worn out severely, while injection of 5Z-7 alleviated that wear in a dose-dependent manner, H&E and toluidine blue staining results indicated that 5Z-7 significantly reversed synovial hyperplasia and cartilage destruction caused by DMM surgery (Fig. 6c). As assessed by OARSI grading scale, DMM rats developed severe OA one month after surgery, while lower dose of 5Z-7 injection group showed significantly lower OARSI grades compared to DMM group, and higher dose of 5Z-7 decreased the grades even more, much closer to normal control group (Fig. 6d). In addition, we detected decreased expression of type II collagen, and increased expression of MMP13 and IL-6 in articular cartilage of DMM rats, which as expected, were reversed by increasing doses of 5Z-7 (Fig. 6e). Together, these results suggested that TAK1 inhibition by 5Z-7 effectively prevented cartilage destruction and synovial inflammation in post-traumatic OA.

## Discussion

Human osteoarthritis (OA) is a degenerative disease of the joint that is among the leading causes of chronic disability^25^. Although great efforts have been devoted into elucidating the molecular mechanisms during OA development, there is still no effective disease-modifying treatment for OA currently. Therefore, further understanding of OA pathogenesis is imperative to investigate potential therapeutic targets for the disease.

Evidences have shown that inflammatory response genes, such as cytokines, chemokines, adhesion molecules and matrix-degrading enzymes, play a significant role in OA pathogenesis^26^, And the expression of inflammatory response genes is mainly controlled by NF-κB and MAPK pathways, which can be triggered by multiple stress-related stimuli including excessive mechanical stress, ECM degradation products and pro-inflammatory cytokines. In the context of OA disease, NF-κB, JNK and p38 MAPK signaling pathways together constitute a complicated regulatory network, and promote OA progression from multiple angles. Therefore, inhibiting either of the above pathways might not be sufficient enough to significantly ameliorate OA, which makes their mutual upstream kinase TAK1 a more attracting therapeutic target. Herein, using primary chondrocytes and synoviocytes derived from rats as well as OA patients, we have provided convincing evidences that inhibition of TAK1 activity by small molecular inhibitor 5Z-7 efficiently suppressed NF-κB, JNK and p38 activation in cells under OA condition.

Since pro-inflammatory cytokine-induced inflammation changes in synovium greatly contributed to OA pathogenesis^27^,^28^, it is reasonable to deduce that OA patients should greatly benefit from biologic approaches that alter the pathologic responses of not only cartilage but also synovium. In our study, we showed that TAK1 inhibition by 5Z-7 significantly modulated the expression of matrix degradation enzymes, ECM protein and pro-inflammatory cytokine in OA chondrocytes as well as synoviocytes. Furthermore, we demonstrated 5Z-7 ameliorated microenvironment homeostasis in a co-culture system consisted of chondrocytes and synoviocytes. We noticed that the regulatory function of 5Z-7 was greater in chondrocytes co-cultured with OA synoviocytes compared to those with normal synoviocytes, indicating that 5Z-7 not only directly acts on chondrocytes, but also affects them through acting on synoviocytes (Fig. 5c). The ECM-related genes expression in chondrocytes co-cultured with normal synoviocytes was also impacted by 5Z-7, which was probably due to the fact that the normal chondrocytes and synoviocytes used in this study were harvested from patients with other joint diseases, which makes them relatively normal, but still different from cells in completely healthy status. More importantly, using a well-established post-traumatic rat model of OA, we showed that TAK1 inhibition significantly prevented not only cartilage destruction but also synovial inflammation. Together, it suggested that 5Z-7 modulates both cartilage and synovium to exert its dual suppressive function towards OA progression.

Recent developments have implicated that in addition to IL-1 and other inflammatory cytokines, enhanced expression of IL-6 plays an important role in OA. A prospective population-based study on a British cohort showed a correlation of higher BMI and elevated serum levels of IL-6 in the development of radiographic knee OA^29^. IL-6 has also been reported to inhibit the expression of type II collagen and induce cartilage collagen breakdown as well as collagenase production^30^,^31^, it has also been associated with hyperalgesia and hypersensitivity in joint tissues^32^. Interestingly, IL-6 was also found to act on sensory neurons and increase their susceptibility, suggesting a potential role of IL-6 in pain propagation in arthritic states^33^. Our results showed that 5Z-7 very potently decreased IL-6 level in a manner much stronger than other ECM-related genes tested, which further illustrated its mechanism of action in ameliorating the symptoms of OA.

Now that we have demonstrated the ability of TAK1 inhibitor 5Z-7 to ameliorate OA using *in vitro* cell model as well as a rat model of OA, future studies need to be focused on assessing the treatment efficacy of 5Z-7 in larger preclinical animal models of OA, as well as its extra-articular or systemic side effects as we cannot dismiss that, and then move forward to clinical trials as was shown for biologic drugs in the use of RA treatment.

In conclusion, our study has provided the first proof-of-concept that TAK1 overexpression in the joint is sufficient enough to induce OA, highlighting the involvement of TAK1 signaling in cartilage homeostasis imbalance, and inhibition of TAK1 enzymatic activity by 5Z-7 prevents osteoarthritic cartilage destruction as well as synovial inflammation by regulating a series of ECM-related genes. On the basis of our results, we suggest that TAK1 is a potential target for OA therapeutic intervention, and 5Z-7 could be the lead compound for relevant drugs.

## Materials and Methods

All methods in this study were performed in accordance with the relevant guidelines and regulations.

### Intra-articular injection of adenovirus in rats

Twenty Sprague-Dawley (SD) rats weighing 80 g were randomly divided into four groups, five rats each group: Normal group, Ad-C group (1 × 10^8^ pfu Ad-ctl), Ad-*Tak1* L (1 × 10^7^ pfu Ad-*Tak1*) group and Ad-*Tak1* H (1 × 10^8^ pfu Ad-*Tak1*) group. The adenovirus was diluted in 50 μL of physiological saline and injected into the articular through the patellar ligament using a 26-gauge needle twice a week. After one month, the rats were sacrificed under anesthesia. Ethical approval was obtained from Animal Care and Use Committee of Peking University Health Science Center. All methods in this study were performed in accordance with the approved guidelines and regulations.

### Induction of DMM and intra-articular injection of 5Z-7

The induction of DMM was conducted as previously described^23^. Three days after surgery, the rats were randomly divided into three groups: DMM (DMSO control) group, DMM + 5Z-7 L (0.5 mg/kg 5Z-7) group and DMM + 5Z-7 H (1 mg/kg 5Z-7) group, five healthy rat joints were used for normal control group. 5Z-7 was diluted in 50 μL of DMSO and injected into the articular twice a week, and continued for one month until the rats were sacrificed.

### Primary chondrocytes and synoviocytes isolation and culture

Primary rat chondrocytes were isolated from the femoral condyle and tibial plateau of SD rats weighing 80 g. Rat articular cartilage was cut into small fragments, followed by digestion with 0.25% trypsin for 30 min and then with 0.2% type II collagenase for 4 hrs at 37 °C. Then cells were suspended in complete medium. OA chondrocytes were isolated from knee cartilage of OA patients, which was obtained from Institute of Sports Medicine, Peking University Third Hospital at the time of joint replacement. Relatively normal human chondrocytes were isolated from knee cartilage of patients with osteochondritis dissecans, with the approval of Human Ethics Committee of Peking University Third Hospital and patients’ written informed consent.

Primary rat synoviocytes and human OA synoviocytes were isolated from the synovium of SD rats and OA patients, respectively. Relatively normal synoviocytes were harvested during second-look arthroscopy from patients who have received ACL reconstruction. The synovium was cut into small fragments, and then digested with 0.2% type I collagenase for 4 hrs at 37 °C. Then cells were suspended in complete medium. All cell lines were maintained in a humidified incubator containing 5% CO_2_ at 37 °C.

### Immunofluorescence analysis

Twenty-four hours after the injection of adenovirus, sections obtained from the articular cartilage of rats were rinsed in PBS and then embedded in O.C.T. Compound. Cryosections (8 μm thick) were mounted onto slides and then incubated with Hoechst 33342 for 5 min. After washed with PBS for three times, the slides were observed under a confocal microscope (FV1000 Olympus IX-81).

### Histological evaluation

The distal portions of the rats’ femurs were cut off and fixed in 4% paraformaldehyde for 48 h at 4 °C. The specimens were then decalcified, trimmed, dehydrated in a graded ethanol series, and embedded in paraffin. Sections (5 μm-thick) were stained with hematoxylin-eosin (H&E) and toluidine blue. Immunohistochemistry (IHC) analysis was performed with type II collagen antibody (novusbio, Littleton, CO, USA), IL-6, MMP13 and TAK1 antibody (Abcam, Camrbidge, MA, USA). The Osteoarthritis Research Society International (OARSI) grading system was used to score the changes in rat cartilage^34^,^35^.

### Scanning electron microscopy

The cartilage of rat knee joints was harvested and fixed immediately in 25% glutaraldehyde at 4 °C for 1 day, dehydrated in a graded ethanol series, and then subjected to critical point drying (liquid CO_2_ at 37 °C) to ensure complete dehydration. The specimens were vacuum-coated with a 5 nm layer of gold in a high-vacuum gold spatter coater and then viewed with a S2500 scanning electron microscope (Hitachi, Tokyo, Japan).

### Nanoindentation assessment

Biomechanical analysis of rat cartilage surface was performed using nanoindentation. Samples were isolated from the central part of rat femoral condyle. Hydration was maintained utilizing a circumfluent PBS solution. All indentations were performed using the TriboIndenter (Hysitron, Minneapolis, MN, USA) with a 400 μm radius curvature conospherical diamond probe tip. A trapezoidal load function was applied to each indent site with loading (10 s), hold (2 s), and unloading (10 s). Indentations were force-controlled to a maximum indentation depth of 500 nm. Meanwhile, the microscopic geomorphology of the indentation zones was captured using micro-scanning apparatus.

### Measurement of cytokine changes in the knee synovial fluid

The synovial fluid inside knee joints was harvested and measured using a biotin label-based rat antibody array (RayBio, AAR-BLG, Ray-Biotech, Norcross, GA, USA). In brief, synovial fluid was first dialyzed with dialysis buffer, labeled with biotin and incubated with arrays overnight. Then the glass slides were incubated with Cy3-conjugated streptavidin for 2 h. Finally, the samples were detected using a Bio-Rad Scanner (Hercules, CA, USA), and the images were analyzed using RayBio analysis tool.

### RNA isolation and real-time PCR analysis

Total RNA was extracted using TRIzol reagent. Isolated RNA was reverse-transcribed and real-time PCR was performed using the Applied Biosystems StepOnePlus Real-Time PCR System (Foster City, CA, USA). The expression level of GAPDH was used as internal control. The relative expression level was calculated by 2^−ΔCT^. The sequences of the RT-PCR primers were shown in Supplementary Table S1.

### Western blotting assay

Cell pallets were lysed with RIPA lysis buffer, resolved by SDS polyacrylamide gel electrophoresis (PAGE) and transferred to PVDF membranes. The membranes were then incubated with corresponding primary antibodies overnight at 4 °C and horseradish peroxidase-conjugated secondary antibodies for 1 hr at room temperature. The membranes were visualized by BIO-RAD ChemiDoc XRS+ system. Anti-phospho-IKKα/β (Ser176/180), anti-phospho-IκBα (Ser32/36), anti-phospho-p38 (Thr180/Tyr182), and anti-phospho-JNK (Thr183/Tyr185) antibodies were obtained from Cell Signaling Technology (Danvers, MA, USA); anti-GAPDH, anti-mouse and anti-rabbit secondary antibodies were purchased from ZSGB-BIO (Beijing, China).

### Cell proliferation assay

Chondrocytes and synoviocytes were seeded in a 96-well plate at 10^4^ cells per well and incubated at 37 °C for 24 hrs. Then increasing doses of 5Z-7 were added into each well. Twenty-four hours later, 10 μL of CCK-8 (Dojindo, Rockville, MA, USA) was added into each well, and one hour later the absorbance was measured at 450 nm using the microplate reader. Background reading of medium was used to normalize the result.

### Co-culture of chondrocytes and synoviocytes

Relatively normal human chondrocytes were seeded into a 6-well plate and incubated for 24 hrs. Meanwhile, relatively normal synoviocytes were seeded into hanging transwell inserts with complete medium and also cultured for 24 hrs. Then each insert was put into each well containing chondrocytes accordingly, medium with or without 5Z-7 was added into each well. After co-culturing for 24 hrs or 48 hrs, total RNA of synoviocytes and chondrocytes was extracted using TRIzol reagent respectively, and followed by reverse-transcription and real-time qPCR.

### Luciferase reporter assay

Cells were seeded in a 12-well plate at 10^5^ cells per well and co-transfected with NF-κB-dependent *firefly* luciferase construct and Actin-*Renilla* luciferase construct. Luciferase activity was quantified 36 hrs after transfection using Dual-Luciferase Reporter Assay System (Promega, Madison, WI, USA). The relative luciferase activity was determined by dividing *firefly* luciferase activity by Actin-*Renilla* luciferase activity.

### OA cartilage explants experiments

Macroscopically intact knee cartilage from OA patients was obtained from Peking University Third Hospital at the time of joint replacement, with the approval of the hospital ethical committee and patients’ written informed consent. Cartilage disk from the same place were cut into 2 mm square pieces, and each piece was cultured in medium supplemented with 1 or 2 μM 5Z-7 for a total of 10 days (medium was changed every 2 days). Then the cartilage explants were fixed, cut into sections and stained with H&E and toluidine blue.

### Statistical analysis

All experiments were performed in triplicates and reproduced independently at least twice. All histological analysis was performed by a “blinded” observer. Data are represented as mean ± SEM. A two-tailed Student’s t-test was used to determine the statistical significances between two groups. A P value of less than 0.05 was considered statistically significant. Data was analyzed using SPSS software.

## Additional Information

**How to cite this article**: Cheng, J. *et al*. Inhibition of transforming growth factor β-activated kinase 1 prevents inflammation-related cartilage degradation in osteoarthritis. *Sci. Rep.* **6**, 34497; doi: 10.1038/srep34497 (2016).

## Supplementary Material

Supplementary Information
