# Supplementary material for: Inhibition of transforming growth factor β-activated kinase 1 prevents inflammation-related cartilage degradation in osteoarthritis
Source: Sci Rep. 2016 Sep 29;6:34497. doi: 10.1038/srep34497 (PMC5041103; doi:10.1038/srep34497)
Supplement: Supplementary Information [file srep34497-s1.pdf]

Title: Inhibition of transforming growth factor β-activated kinase 1 prevents inflammation-related cartilage degradation in osteoarthritis

Authors: Jin Cheng, Xiaoqing Hu, Linghui Dai, Xin Zhang, Bo Ren, Weili Shi, Zhenlong Liu, Xiaoning Duan, Jiying Zhang, Xin Fu, Wenqing Chen, Yingfang Ao*

Supplementary Figure S1

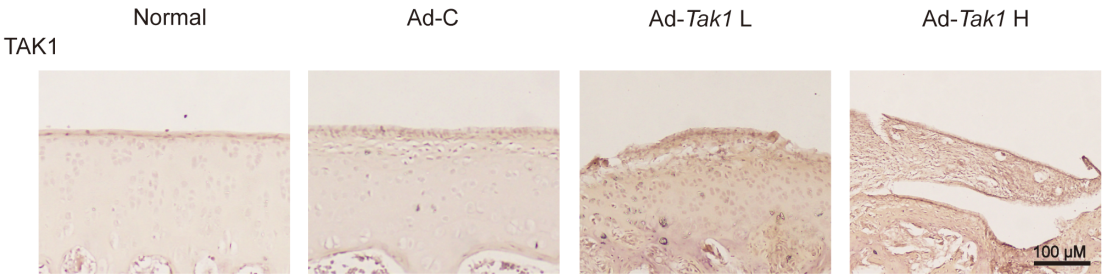

Figure S1 IHC staining of TAK1 for rat cartilage from each group. Scale bar, 100 μm.

Supplementary Figure S2

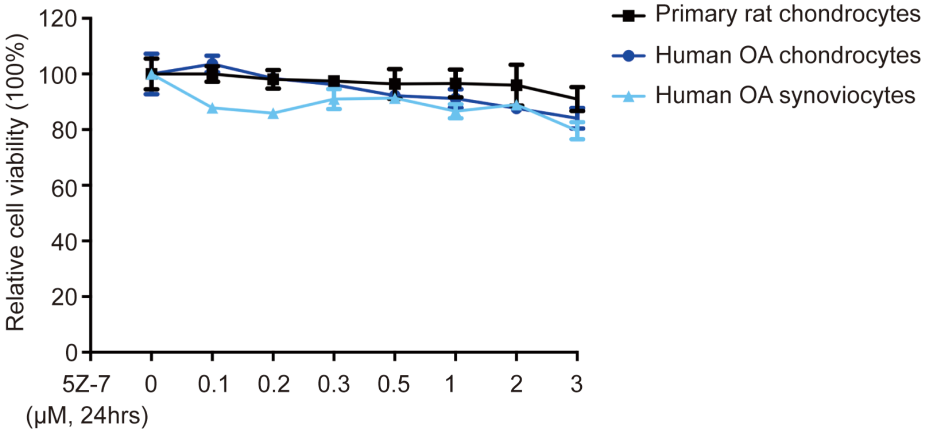

Figure S2 TAK1 inhibitor 5Z-7 exhibited no significant cytotoxity to chondrocytes and synoviocytes. The relative cell viability of primary rat chondrocytes, human OA chondrocytes and synoviocytes treated with increasing doses of 5Z-7. Data were presented as mean ± SEM.

Supplementary Figure S3

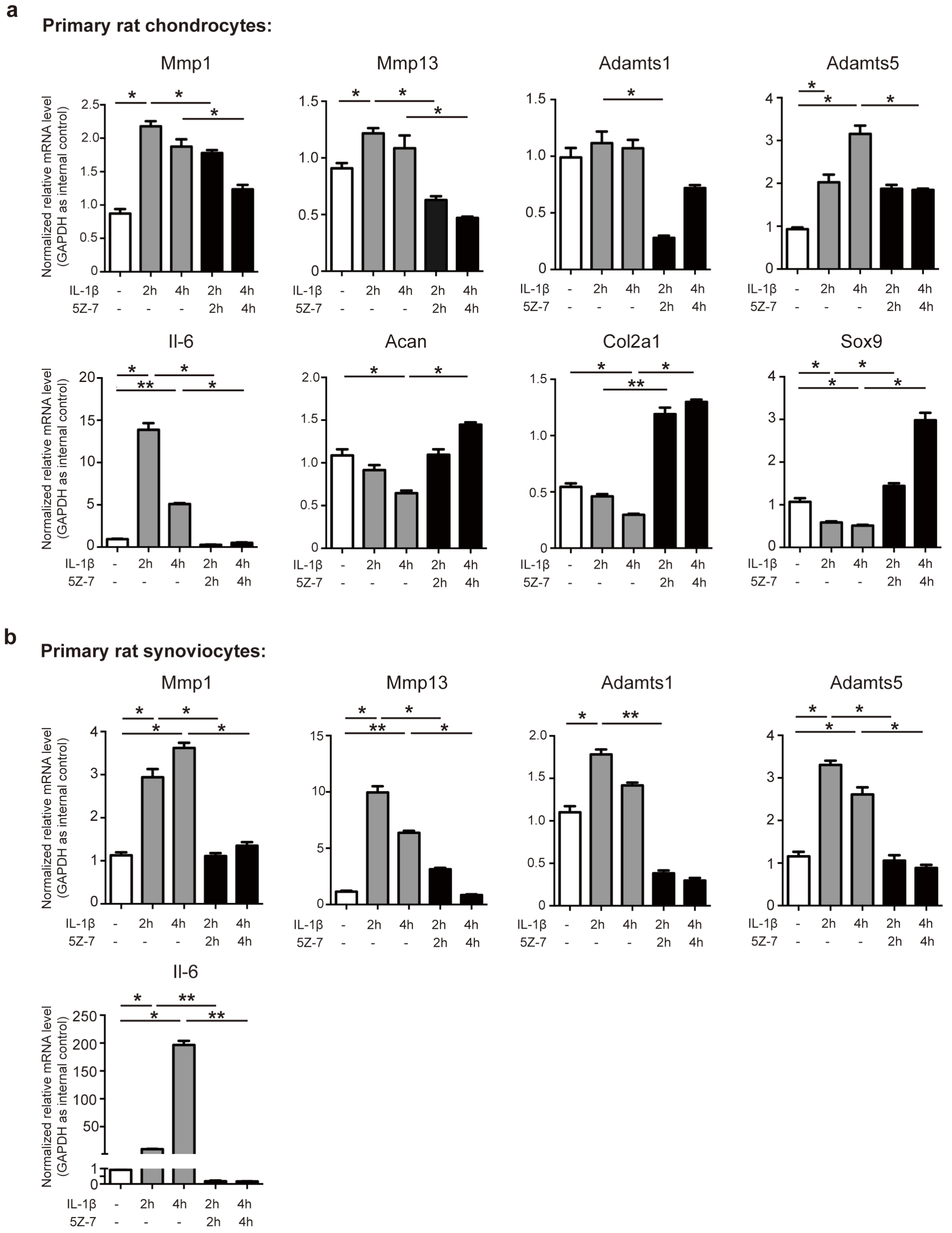

Figure S3 5Z-7 inhibited IL-1β-induced expression changes of ECM-related genes in chondrocytes and synoviocytes. (**a, b**) The mRNA expression level of classic ECM-related genes in (**a**) primary rat chondrocytes and (**b**) primary rat synoviocytes treated with IL-1β at indicated time points with or without 5Z-7. *$P< 0.05$, **$P<0.01$.

Supplementary Figure S4

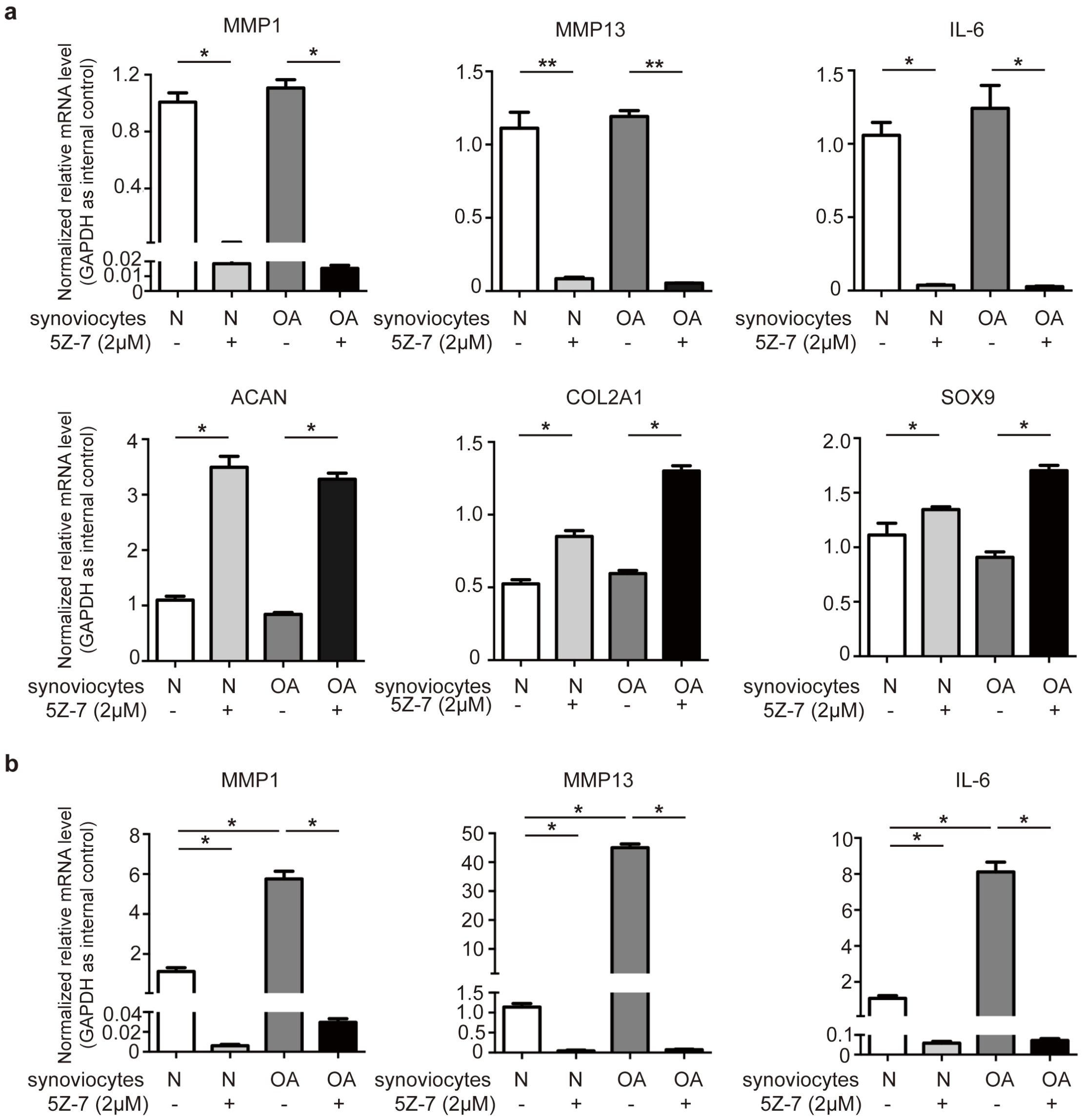

Figure S4 TAK1 inhibition by 5Z-7 modulated microenvironment homeostasis in the co-culture system. (**a, b**) The mRNA expression level of classic ECM-related genes in (**a**) chondrocytes and (**b**) synoviocytes within the co-culture system after 48 hrs. *$P<0.05$, **$P<0.01$.

Supplementary Table S1: The real-time primer sequences for ECM-related genes in rat and human.

Primer sequences for the detection of target genes expression in rats

Gene name	Forward (5'-3')	Reverse (5'-3')
**Mmp1**	GCAGTGTGACACACATTGAC	GATAACCTGGATCCATGGAC
**Mmp13**	CTGCGGTTCACTTTGAGGAC	ACAGCATCTACTTTGTCGCC
**Adamts1**	GCCTACATGGTCACATCATTCCT	TGGCGGTTGGCATCGTA
**Adamts5**	CACGACCCTCAAGAACTTTTGC	TCACATGAATGATGCCCACATAA
**Il-6**	AAGCCAGAGTCATTCAGAGC	TTAGCCACTCCTTCTGTGAC
**Acan**	CCACTGGAGAGGACTGCGTAG	GGTCTGTGCAAGTGATTCGAG
**Col2a1**	CACCGCTAACGTCCAGATGAC	GGAAGGCGTGAGGTCTTCTGT
**Sox9**	AGGAAGCTGGCAGACCAGTA	ACGAAGGGTCTCTTCTCGCT
**Gapdh**	GCAAGTTCAACGGCACAG	GCCAGTAGACTCCACGACA

Note: The annealing temperature of the primers is 60℃.

Primer sequences for the detection of target genes expression in human

Gene name	Forward (5'-3')	Reverse (5'-3')
**MMP1**	TGAACTCGGCCATTCTCTTG	AACGTCCATATATGGCTTGG
**MMP13**	ACTGAGAGGCTCCGAGAAATG	GAACCCCGCATCTTGGCTT
**ADAMTS1**	GTGATCGCATCATAGACTCC	TGGCTCCAGTTGGAATTGTG
**ADAMTS5**	GAACATCGACCAACTCTACTCCG	CAATGCCCACCGAACCATCT
**IL-6**	TTCGGTACATCCTCGACGGCATC	CAGCTCTGGCTTGTTCCTCACTAC
**ACAN**	ACTCTGGGTTTTCGTGACTCT	ACACTCAGCGAGTTGTCATGG
**COL2A1**	CCAGATGACCTTCCTACGCC	TTCAGGGCAGTGTACGTGAAC
**SOX9**	AGCGAACGCACATCAAGAC	CTGTAGGCGATCTGTTGGGG
**GAPDH**	GCACCGTCAAGGCTGAGAAC	TGGTGAAGACGCCAGTGGA

Note: The annealing temperature of the primers is 60℃.
